# Development of an implementation science informed “Test Evidence Transition” program to improve cancer outcomes

**DOI:** 10.3389/frhs.2024.1328342

**Published:** 2024-04-18

**Authors:** Kate E. Hamilton-West, Alexandra Feast, Natalie A. Masento, Brian Knowles, Claire Sloan, Luke Weaver

**Affiliations:** Social and Behavioral Research Team, Evidence and Implementation Department, Policy Information and Communications Directorate, Cancer Research UK, London, United Kingdom

**Keywords:** cancer, implementation science, behavioral science, evaluation, implementation research, quality improvement

## Abstract

**Introduction:**

Translation of cancer research into practice takes around 15 years. Programs informed by implementation science methods and frameworks offer potential to improve cancer outcomes by addressing the implementation gap.

**Methods:**

We describe the development of a Test Evidence Transition (TET) program which provides funding and support to health system delivery teams and project design and evaluation partners working together to achieve three objectives: Test innovations to support optimal cancer pathways that transform clinical practice; Evidence the process, outcome, and impact of implementation; and work with strategic partners to ensure the Transition of best practice into effective and equitable adoption across UK health systems.

**Results:**

Phase 1 launched in April 2023. Teams with the capability and motivation to implement evidence-based pathway innovations were identified and invited to submit expressions of interest. Following peer-review, teams were supported to develop full proposals with input from academics specializing in health services research, evaluation, and implementation science. Projects were selected for funding, providing an opportunity to implement and evaluate innovations with support from academic and health system partners.

**Conclusions:**

TET aims to improve cancer outcomes by identifying and addressing local-level barriers to evidence-based practice and translating findings into consistent and equitable adoption across health systems. Phase 1 projects focus on pathway innovations in diagnosis for breast and prostate cancer. We are now launching Phase 2, focusing on colorectal cancer.

## 1 Introduction

Cancer is one of the world's greatest challenges. In the UK, almost 1 in 2 people will get cancer in their lifetime (1). Beating cancer requires sustained and targeted work to drive scientific discovery and translate this into benefits for people affected by cancer (2). However, translation of evidence into practice is complex and challenging: a 2021 study of cancer prevention, screening, treatment, and survivorship research found time to translation averaged around 15 years. The study noted differences in the speed of implementation by race/ethnicity highlighting the role of inequitable access and use of health services in driving cancer health disparities (3). The slow and uneven adoption of evidence into practice creates unwarranted variation in cancer care delivery and deficits in quality and safety (4).

There are multiple factors underlying this “implementation gap”. For example, Mitchell and Chambers (5) highlight that efficacy studies do not usually provide information about barriers and enablers of implementation or the effects of individual and organizational context on intervention efficacy; efficacy studies are also not generally designed to manualize an intervention for use in routine practice, confirm the nature and extent of intervention adaptation that is permissible while preserving efficacy, or address sustainability of the intervention in delivery settings. Researchers further highlight that Quality Improvement (QI) initiatives are often used to increase uptake of Evidence Based Interventions (EBIs) but these tend to be local in focus and not designed to enable consideration of the generalizability of findings or understanding of the mechanisms underlying practice change, while reports on the impact of QI often provide limited detail on the strategies used or extent of user engagement (5, 6). Introducing innovations into care delivery settings, both within the cancer context and more widely is often opportunistic rather than systematically planned and may be based on untested assumptions (e.g., relating to the effectiveness and acceptability of the innovation, the skills and resources needed to deliver it safely and effectively and fit with the wider care pathway) (5, 7, 8). Such problems may be compounded by a lack of capacity for research and innovation among those in frontline healthcare roles, with high demands of clinical services and no protected time for research cited as frequent barriers (9).

Service innovation projects also tend to pay insufficient attention to the organizational, financial, and human resources needed to scale-up innovations beyond the local level. Consequently, managers responsible for roll out at regional or national level are faced with the challenge of implementing the innovation with few resources in health systems that may be characterized by weak capabilities and multiple pressing priorities (10).

## 2 The role of implementation science

The gap between identification of evidence-based innovations and translation into practice has led to development of the field of implementation science (also referred to as dissemination science improvement science, and implementation research), which Eccles and Mittman (11) define as “the scientific study of methods to promote the systematic uptake of research findings and other evidence-based practices into routine practice, and, hence, to improve the quality and effectiveness of health services and care” (p1). With interdisciplinary underpinnings, implementation science involves a theory-driven approach that explicitly examines the link between an intervention and an outcome and seeks to explain why the intervention worked or failed in a particular setting, as well as considering the influence of context on health care professional and organizational behaviors (5, 12).

In addition to facilitating the implementation of a specific intervention, the systematic approach underpinning implementation science research can lead to creation of generalizable knowledge surrounding methods for the sustainable implementation of interventions across studies and settings (12). This includes understanding the most effective techniques to improve the dissemination of evidence; incorporate new discoveries into clinical care delivery (preserving intervention fidelity and sustainability) and intervene on the determinants of successful and failed clinical implementation (5). Implementation science theories, models and frameworks are used to inform evaluation of barriers and facilitators, identify stakeholders, guide the selection of implementation strategies, and anticipate and manage implementation failures. They also provide a framework to measure the effectiveness of implementation and identify factors that should be considered to achieve sustainability (5). There is increasing recognition that implementation science has a vital role to play in transforming the delivery of evidence-based cancer care, addressing persistent disparities and driving improvements in patient outcomes (5, 12). The growing global burden of cancer (13) points to a need to accelerate these efforts, including targeted initiatives to fund and support the development, refinement and application of implementation science methods and approaches (6, 14, 15).

## 3 The test evidence transition program

Test Evidence Transition (TET) is a multi-phase program of commissioned activity delivered by Cancer Research UK (CRUK), the world's leading independent cancer charity dedicated to saving lives through research, influence, and information. TET was developed to support CRUK's strategic “Translate” objective, which focuses on addressing the implementation gap by working “with health systems to make sure that best practice is rolled out consistently, effectively and equitably across health systems to benefit everyone” (p20) (2). In line with this objective, the program aims to:

• *Test* service innovations to support the delivery of optimal cancer pathways at a local level.
• *Evidence* the process, outcomes, and impact of implementation at a local level, focusing on identified metrics that will drive adoption; and
• Work with strategic partners to enable the effective and equitable *Transition* of identified best practice into mainstream practice across the health system.

TET builds on the former Accelerate, Co-ordinate, Evaluate (ACE) Program which was developed in collaboration between CRUK, Macmillan Cancer Support and NHS England to meet an identified need for published evidence on the impact of service innovations in real world contexts (16). A “Wave 1” cohort of 60 projects added to the evidence base for known innovations in the priority areas of lung and colorectal cancer pathways, patients presenting with vague symptoms, and uptake of bowel screening. The “Wave 2” cohort of five pilot projects provided proof of concept evidence for novel rapid diagnostic pathways for patients presenting with *nonspecific but concerning symptoms* indicative of possible cancer, a more precise term that evolved iteratively during the “Wave 1” cohort from vague symptoms (17).

A Realist Evaluation of projects supported in Wave 1 found that the most important enabling conditions (“contexts”) identified were the prevailing organizational culture (history of service improvement at this scale, with a proactive approach to developing services and change management) and commitment to quality improvement. Being part of a high profile, national initiative was also important. The mechanisms that emerged as enabling implementation included good quality project management, clinical leadership and engagement and communication within and between partner organizations. The extent to which these were present, as well as the extent to which service improvements were normalized, or incorporated into the working practice of user and providers varied across projects (18). Interviews with project teams also revealed that having a network of committed people was vital for both initiating and sustaining change, while understanding stakeholders' emotional responses to change helped mitigate emergent challenges (19). This multiplicity of intermediate conditions, acknowledged in the ACE Theory of Change model (20), while depicting the complexity of change, also enabled ACE to influence through a variety of routes in pursuit of its strategic goal, sustaining health systems innovation.

TET aims to build on the successes of the ACE program by ensuring these elements are in place at the planning stage, applying implementation science models, frameworks, and approaches to identify barriers and facilitators of implementation, promote the systematic uptake of evidence-based innovations and create the conditions necessary for effective and equitable scale up across health systems. Key program elements are described below. Table 1 links these to implementation science strategies as defined by Expert Recommendations for Implementing Change (ERIC) initiative (21) ERIC has been highlighted as a useful resource for embedding implementation science in cancer improvement initiatives (5).

**Table 1:** TET program strategies and practical application.

TET objectives	Barriers identified	Strategy^a^	Practical application
**Test** service innovations to support the delivery of optimal cancer pathways at a local level.	Lack of capacity for service innovation	Identify and prepare champions	TS
		Assess for readiness and identify barriers and facilitators	TS
		Obtain formal commitments	TS
		Access new funding	TS
		Recruit, designate, and train for leadership	TS
	Lack of organisational support for service innovation	Involve executive boards	ML
		Inform local opinion leaders	ACM
	Lack of access to methodological expertise/high quality project management	Work with educational institutions	ML
		Develop academic partnerships	DTR
		Develop and implement tools for quality monitoring	DTR
	Lack of support for navigating the challenges involved in implementation, evaluation and scale-up/Lack of access to high quality project management	Build a coalition	ACM
		Centralize technical assistance	ACM
		Facilitation	ACM
		Provide ongoing consultation	ACM
		Use advisory boards and workgroups	ACM
**Evidence** the process, outcomes, and impact of implementation at a local level, focusing on identified metrics that will drive adoption	Insufficient evidence on impact in real world contexts	Use data experts	ML
		Audit and provide feedback	ACM
	Insufficient evidence on acceptability of innovations/extent of user engagement	Involve patients/consumers and family members	ML
	Insufficient evidence on skills and resources needed to deliver innovations safely and effectively	Use an implementation advisor	CD
	Insufficient evidence on how and to what extent innovations can be adapted while preserving efficacy	Purposely reexamine the implementation	CD
		Promote adaptability	CD
		Tailor strategies	CD
Work with strategic partners to enable the effective and equitable **Transition** of identified best practice into mainstream practice across the health system.	Implementation efforts may not be designed to inform adoption beyond the local level	Create a learning collaborative	ACM
		Capture and share local knowledge	ACM
		Stage implementation scale up	PP
		Organize clinician implementation team meetings	PP
		Promote network weaving	PP
		Visit other sites	PP
	Implementation efforts may not be designed to manualise innovations for use in routine practice	Develop a formal implementation blueprint	SS
		Develop an implementation glossary	SS
		Develop and implement tools for quality monitoring	SS
		Develop educational materials	SS
		Distribute educational materials	SS
		Use mass media	SS

^a^ Strategies are drawn from the Expert Recommendations for Implementing Change (ERIC) (21). Please refer to the original source for full concept definitions.

TS, Team Selection; ML, Multidisciplinary Leadership; ACM, Active Commissioner Model; DTR, Development of Tools and Resources; PP, Program Phasing; CD, Co-design; SS, Support for Scale-up.

### 3.1 Multidisciplinary leadership

TET is led by CRUK's Social and Behavioral Research Team in close collaboration with teams across the Evidence and Implementation Department, ensuring that the program design is informed by a detailed understanding of the cancer research and care landscape, underpinned by data and evidence and supported by strong stakeholder relationships (including health systems leaders, academic experts, clinicians, patients and public). This multidisciplinary leadership team is responsible for the program design, including the identification of implementation strategies relevant to TET objectives and their translation into practical application in the organizational context (e.g., considering fit with wider strategic objectives and resources available).

### 3.2 Team selection

Health systems delivery teams with the capability and motivation to implement and scale an innovation are invited to apply for funding and support. Applicants are asked to describe the team's ability and experience in delivering, mobilizing, implementing, and spreading a service innovation. Applications must also demonstrate appropriate stakeholder engagement and involvement (including patients and the public), understanding of relevant governance frameworks, consideration of potential risks to project delivery and how these may be addressed. These criteria are intended to ensure that the enabling conditions identified from the former ACE program (18, 19) are in place from the start. Consistent with the program objectives, applications must also demonstrate consideration of health inequalities. Applications are subjected to peer review and funding panel discussion prior to selecting successful teams.

### 3.3 Active commissioner model

The program adopts an “active commissioner” model, in which projects selected for funding receive ongoing support throughout the process of designing, implementing, evaluating, and scaling innovations. This includes support from the TET program team and stakeholders (described above), as well as project design and evaluation partners—experts in relevant research and evaluation methodologies funded by CRUK to support the project teams and wider program. The program team also has expertise in methodologies relevant to TET, including health services research, evaluation, behavioral science, implementation science, quantitative and qualitative methods.

CRUK supports the delivery of projects using management approaches including an initiation meeting, project initiation document, regular monitoring and progress reports, a communications and dissemination plan, comprehensive critical appraisal and coproduction of project outputs including quality assurance and sign-off. Strategic oversight and monitoring of risks and progress are provided via regular steering group meetings at both the project and program level.

### 3.4 Program phasing

In line with the program objectives, TET focuses on innovations which are already proven to be effective, with an existing evidence base, that need to be implemented more effectively and equitably. Each phase of the program has a specific focus (e.g., geographical region, cancer type), identified as a key priority for CRUK, ensuring resources are directed toward activities with the greatest potential to improve cancer outcomes.

Phase 1 projects launched in April 2023, with a focus on the UK's devolved nations. This phase of the program provides funding and support to three teams in Scotland and Wales implementing and evaluating innovations to improve the timelier diagnosis of breast and prostate cancer. Table 2 provides an overview of the three Phase 1 projects.

**Table 2:** Summary of phase 1 projects.

Organizations	Project title	Aim	Design	Implementation science model/Framework/Approach
NHS Forth Valley, Scotland; University of Stirling and the National Centre for Sustainable Delivery; NHS Scotland. UK.*	Implementing fast-track access from primary care to a breast assessment clinic for patients presenting with a breast lump: an evaluation and scalability assessment.	To introduce, evaluate and assess the scalability of a change in pathway in NHS Forth Valley to allow fast-track access to assessment clinics for symptomatic patients with a breast lump to support earlier breast cancer diagnosis	A mixed methods approach, collecting and analyzing qualitative and quantitative data. A hybrid effectiveness-implementation design (22) will allow for the identification of potential improvement within a complex system. A naturalistic case study design will be used to explore context and process (23).	Design is guided by the Evidence Integration Triangle (EIT) (24) and the evaluation uses Theory of Change (ToC) (25).
NHS Fife, Scotland; University of Stirling; the National Centre for Sustainable Delivery; NHS Scotland. UK.^a^	Improving the suspected prostate cancer diagnosis pathway: a hybrid effectiveness implementation evaluation of an advanced clinical nurse specialist-led model in Fife.	To develop, implement and evaluate an Advanced Clinical Nurse Specialist-led prostate cancer diagnostic model in NHS Fife, to improve the process from referral to diagnosis up until decision to treat	As above.	As above.
Hywel Dda University Health Board, Wales; Swansea University; the Wales Cancer Network, NHS Wales. UK.	Development of a Model Prostate Cancer Diagnostic Pathway	To develop, pilot and evaluate the Model Prostate Cancer Diagnostic Pathway within the service, to shorten time from referral to diagnosis (and potentially time to definitive treatment).	A mixed methods approach, collecting and analyzing qualitative and quantitative data. The design incorporates a whole systems co-produced approach including implementation review, qualitative interviews, continuous data review and an economic model to estimate the cost and consequences of the novel pathway compared to the usual standard pathway.	A Realist Evaluation (RE) approach (26) has been adopted. Plan plan-do-study-act cycles will be used (27).

^a^ Please refer to the published protocol paper for further detail on the two projects in Scotland (28).

Further detail in relation to the two projects in Scotland is provided in the recently published protocol paper (28). Phase 2 will have a UK-wide focus, supporting teams to implement and evaluate innovations with the potential to improve timely detection and diagnosis of colorectal cancer.

Projects receive funding and support for a period of 18 months to two years. This phased approach allows for learnings from each phase to inform the design of subsequent phases, while overlap between the funded projects allows for cluster meetings, bringing teams together to share learnings. Program phasing is also intended to facilitate analysis of data and insights across the funded projects, drawing out considerations relevant to specific contexts, populations, and innovations.

### 3.5 Co-design

An early learning from Phase 1 was that health systems delivery teams with the capability and motivation to implement service innovations do not necessarily have established relationships with academics specializing in research and evaluation methodologies, which presents a barrier to developing funding applications. For Phase 2, we therefore adopted a different approach. Health systems delivery teams may apply without identifying academic collaborators or proposing a detailed methodology. Once health systems delivery teams are selected for funding, projects will then be co-designed with input from the project design and evaluation partners, TET program team and wider stakeholders.

The co-design process is intended to ensure:

• A robust methodology, informed by relevant frameworks (e.g., implementation science, behavioral science, health services research, service improvement and evaluation)
• A realistic and deliverable project design, informed by an understanding of the health service delivery context and factors influencing evaluability (e.g., availability of data and resources)
• Collection and analysis of qualitative and quantitative (including economic) data required to evidence the implementation process and outcomes and build a case for Transition.
• Appropriate patient, public and stakeholder involvement in the co-design process and throughout (including local and national stakeholders relevant to Transition)
• Robust research governance and project management processes (e.g., supporting teams to submit projects for ethical review, monitor project timelines, milestones, and key deliverables, identify risks and mitigations)
• Close collaboration with CRUK's Test Evidence Transition program team throughout, including regular communication (e.g., emails, virtual/face-to-face meetings), attendance and reporting to steering group meetings (e.g., updates on activities/timelines, risks/mitigations) and co-authoring reports, publications, and presentations.

Project design and evaluation partners will continue to work closely with health systems delivery teams throughout the implementation and evaluation period. Approaches may be adapted to address barriers and leverage facilitators identified as the projects progress.

### 3.6 Development of tools and resources

In addition, we have commissioned a team of academics to co-create a suite of resources that will assist health systems delivery teams in planning, taking forward and scaling up innovation and evaluation projects. These will include, for example, guidance on developing logic models and monitoring and evaluation frameworks, involving patients and the public and engaging effectively with stakeholders. These resources will be developed with input from the health systems delivery teams, project design and evaluation partners, TET program team and wider stakeholders, including patient and public involvement.

### 3.7 Support for scale-up

A central objective of the TET program is the transition of innovations beyond their site of origin, so that these can be successfully adopted and sustainably scaled across the healthcare system, outlining the routes to scale and the wider-system conditions required for transformative spread. In addition to outputs outlined above, projects (and analysis of program level data) will also deliver:

• Evaluation protocols allowing for innovations to be reproduced at other sites, including assessing fidelity and identifying adaptations required.
• High-quality case-studies with evidence of how and why the innovation has worked at a local level which informs sustained change; detailing the impact of the service innovation compared to the current patient pathway and any resulting impact on improving cancer outcomes; the impact of the innovation on cancer services and how health systems may need to be adapted to deliver more system-wide benefits.
• A detailed assessment of the sustainability of each innovation, supporting the development of a program scalability and transition plan.
• A cost-effectiveness and affordability analysis for each innovation, incorporating any identified direct cost-savings and an assessment of the health economic impact.
• Wider financial analysis capable of producing an influential business case, including cost of transition for commissioners and health system leaders.
• A demonstration of how the innovation ensures equitable access to improve both patient experience and measurable cancer outcomes.
• A demonstration of how acceptable the innovation is to patients and their support networks, and to healthcare professionals, and how these groups are involved in the design, implementation, and ongoing monitoring.
• Implementation handbook including “cheat sheet” identifying barriers and enablers to change which are non-site specific.
• Any necessary training and service planning guides.

Active engagement with local and national level stakeholders throughout the program phases is essential for creating the conditions for scale-up (10, 18).

## 4 Conclusions

The purpose of this paper is to illustrate how implementation science has been incorporated into the design and delivery of a program of commissioned activity which aims to address the slow and uneven adoption of evidence-based practice in cancer care. This program has been developed in the context of recognition among the healthcare community of the significant global burden of cancer and urgent need to address cancer inequalities (13) as well as recognition by researchers and research funders that targeted work is needed to address the implementation gap in cancer care, harnessing methods and approaches from the field of implementation science (5, 6, 12, 14, 15).

TET harnesses implementation science in several ways: by integrating evidence-based implementation strategies into the program design; requiring funded projects to adopt implementation science models, frameworks, and approaches; and involving experts in implementation science throughout the process of peer-review, team selection, co-design, ongoing monitoring and critical appraisal, development of tools and resources (to assist health systems delivery teams in planning, taking forward and scaling up innovation and evaluation projects), and co-production of project outputs (to disseminate findings and permit transition of innovations beyond their site of origin). TET goes beyond the remit of traditional research funding programs by adopting an “active commissioner model” in which funded teams are supported to navigate the complex challenges involved in implementation, evaluation and scale-up, drawing on input from CRUK's multidisciplinary leadership team and wider stakeholders. Program phasing is designed to enable shared learning between teams, as well as analysis of data and insights across projects, drawing out considerations relevant to specific contexts, populations, and innovations.

A limitation of this program is that we are only able to select health system delivery teams with the capability and motivation to take forward innovation projects, which means that we will not capture insights from teams that are either unable or unwilling to engage in this type of program. This is a decision we considered carefully when designing the program selection criteria, as these teams are arguably in even greater need of support. However, lack of motivation and capability to engage in research and innovation in the context of the UK National Health Service often stem from a combination of high demands of clinical service provision and lack of protected time; these are systemic problems, which lie beyond the influence of this program and require a system-based approach involving all components of the research ecosystem (policy, funding, training, academia, healthcare organizations, government bodies) (9, 29). We also considered that such pressures may be exacerbated by adding further unrealistic demands to already overburdened teams—as such, selection criteria were determined by ethical and practical considerations as well as theoretical.

We will continue to report on progress and outcomes as this program moves forward. Insights from funded projects will also be disseminated by project teams, including submissions to peer-reviewed journals. Ongoing engagement with the academic and healthcare communities will be important for ensuring that the program remains grounded in rigorous evidence and findings feedback to inform both healthcare improvement efforts and the wider implementation science literature.

## Data Availability

The original contributions presented in the study are included in the article/Supplementary Material, further inquiries can be directed to the corresponding author.
